# Inactivation Mechanism of *Escherichia coli* Induced by Slightly Acidic Electrolyzed Water

**DOI:** 10.1038/s41598-017-06716-9

**Published:** 2017-07-24

**Authors:** Zhangying Ye, Shuo Wang, Tao Chen, Weishan Gao, Songming Zhu, Jinsong He, Zhiying Han

**Affiliations:** 1 School of Biosystems Engineering and Food Science, Zhejiang University, Hangzhou, 310058 China; 2 School of Environmental & Resource Sciences, Zhejiang University, Hangzhou, 310058 China; 3 College of Food Science and Technology, Yunnan Agricultural University, Kunming, 650201 China

## Abstract

Foodborne disease outbreak caused by food microbiological contamination is a serious public health problem. Slightly acidic electrolyzed water (SAEW), a new ultra-high effect and wide-spectrum disinfectant that is colourless, odourless, and harmless to humans and the environment, is directly used on food surfaces in Japan and America. However, the underlying inactivation mechanism remains unknown. In this study, biochemical and cellular changes were observed to investigate the bactericidal mechanism of SAEW against *Escherichia coli* (*E. coli*). The results indicated that SAEW with a pH of 6.40, an oxidation-reduction potential (ORP) of 910 mV, an available chlorine concentration (ACC) of 60 mg/L, and a volume ratio of 20:1, produced the most effective sterilization action. A fluorescence-based live-dead assay was further used to demonstrate the sterilized effect and the cell esterase activity damage caused by SAEW. During the observation period, within 10 min, the cell morphology changed, which was characterized by cell expansion, cell elongation and increased membrane permeability. Meanwhile, reactive oxygen substances (ROS) were released in the bacterial cells. *E. coli* inactivation and apoptosis induced by SAEW were observed. Our findings illustrate that the bactericidal effects of SAEW against *E. coli* occurred through cellular and biochemical mechanisms of cell necrosis and apoptosis.

## Introduction

Foodborne diseases have continued to be a widespread and growing health problem, in both developed and developing countries^1^. A research study estimated that each year, 31 major pathogens that are acquired in the United States cause 9.4 million episodes of foodborne illness^2^. Developing countries, which bear the brunt of the problem due to the presence of a wide range of foodborne diseases, are even more alarming^3^. Each year, almost 2.2 million people, mostly children, die from diarrhoeal diseases in developing countries. A considerable proportion of these diseases are probably transmitted through unsafe food^4^. In China, pathogenic microorganisms have been reported to be responsible for over 50% of foodborne illnesses since 2006^5^.

*Escherichia coli*, which is widely distributed in intestinal environments^6^, have been recognized as a cause of serious clinical illnesses and mortality in foodborne disease outbreaks that involve an enormous variety of foods^7^. As a vital treatment process, effective sterilization and disinfection of food play an important role in controlling disease outbreaks. Chlorine^8^, hydrogen peroxide^9^, ozone^10^, and organic acids^11^ are chemical sanitizers that are frequently used in the food industry to produce high-quality, microbiologically safe food for human consumption. Nevertheless, it is forbidden to use some chemical sanitizers in several European countries because of the potential formation of carcinogenic halogenated disinfection by-products^12^, which do harm to the environment as well as to human health. In recent years, there have been many investigations on food decontamination approaches, which have focused on the search for alternative sanitizers based on ensuring food quality and safety^13^.

**Figure 1:**
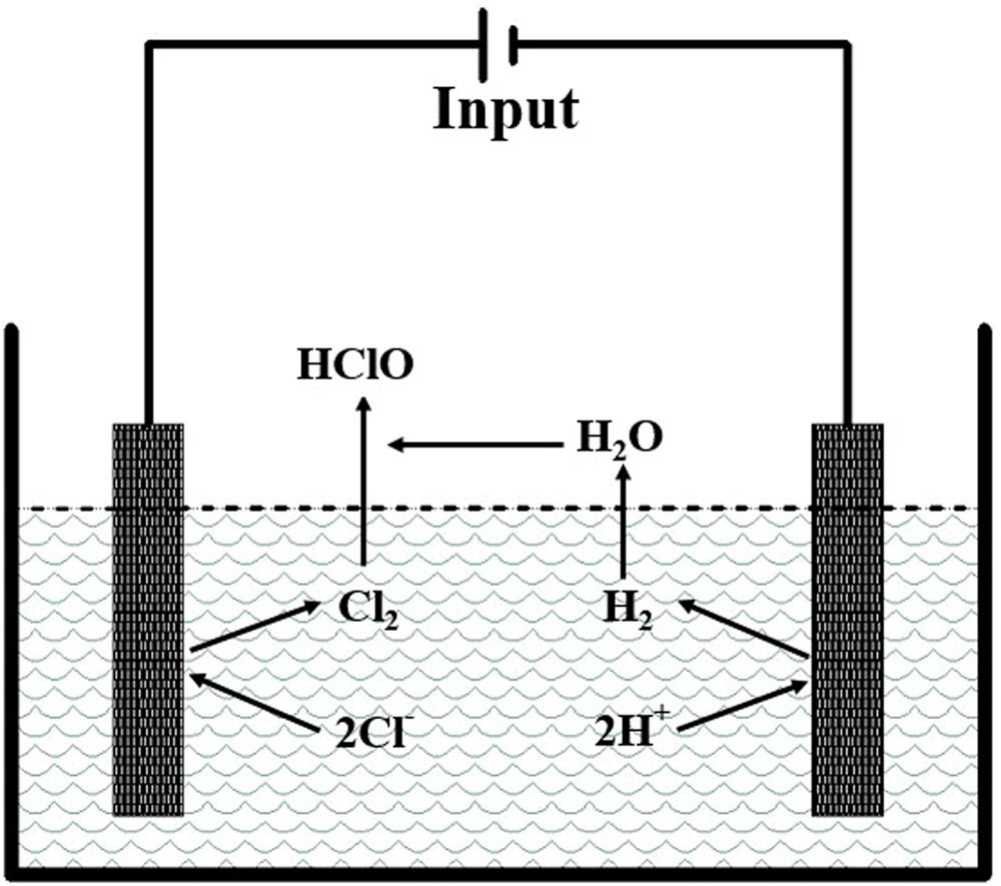
Schematic diagram of a slightly acidic electrolyzed water generator

Slightly acidic electrolyzed water (SAEW) is a novel disinfectant produced by the electrolysis of dilute sodium chloride or hydrochloric acid solutions or both in an electrolytic cell without a separating membrane. Its high bactericidal effect is due to the available chlorine compounds, including ClO^−^, HClO, and Cl_2_^14, 15^. A schematic representation of a SAEW generator is shown in Fig. 1.

The main reaction equations are described as below:

Anode side:

1 $${{\rm{H}}}_{2}{\rm{O}}\to 1/2{{\rm{O}}}_{2}+2{{\rm{H}}}^{+}+2{{\rm{e}}}^{-}$$

2 $$2{{\rm{Cl}}}^{-}\to {{\rm{Cl}}}_{2}+2{{\rm{e}}}^{-}$$

3 $${{\rm{Cl}}}_{2}({\rm{aq}})+{{\rm{H}}}_{2}{\rm{O}}\leftrightarrow {\rm{HClO}}+{\rm{HCl}}$$

Cathode side:

4 $$2{{\rm{H}}}_{2}{\rm{O}}+2{{\rm{e}}}^{-}\to 2{{\rm{OH}}}^{-}+{{\rm{H}}}_{2}$$

The main effective chlorine compound form in SAEW (pH 5.0–6.5) is hypochlorous acid (HClO; 97%), which has strong antimicrobial activity^16^. As a sanitizer, hypochlorous acid is 80 times more effective than an equivalent concentration of the hypochlorite ion (ClO^−^) for inactivating *Escherichia coli* ^17^. The application of SAEW at a near-neutral pH minimizes human health and safety issues from Cl_2_ off-gassing while maximizing the utilization of the hypochlorous acid species^18^. Many studies have investigated the significant effectiveness of the antimicrobial activity of SAEW against different foodborne pathogens, including *Listeria monocytogenes, E. coli* O157:H7, *Staphylococcus aureus*, *Salmonella typhimurium*, *Campylobacter jejuni* and *Vibrio parahaemolyticus* ^14, 19–22^. Meanwhile, many studies have demonstrated the potential use of SAEW as an alternative sanitizer to reduce microbial contamination on vegetables^23–25^; cutting boards^26^; poultry^27^ and sea foods^20, 28^. At the same time, SAEW causes less irritation on the human body and less corrosive effects on food preparation appliances^29, 30^. It was identified as a legal permitted food detergent that can be directly used on food surfaces in Japan and America^31, 32^.

To date, abundant reports of the antibacterial mechanism of SAEW were focused on the physic and chemical properties on SAEW itself. There have been very few studies on the physiological and biological changes of the bacteria by using SAEW. Biochemical and morphological hallmarks of apoptosis and necrosis are popular in eukaryotic cell. Recently, the characteristic phenomena of apoptosis was also observed in *E. coli* which induced by different kinds of bactericidal antibiotic treatment^33^. In this experiment, we devoted to study the cellular and biochemical properties in *E. coli* induced by SAEW, which may illustrate the deep-seated disinfection mechanisms of SAEW.

## Results

### The viability of *E. coli* was inhibited by SAEW

**Figure 2:**
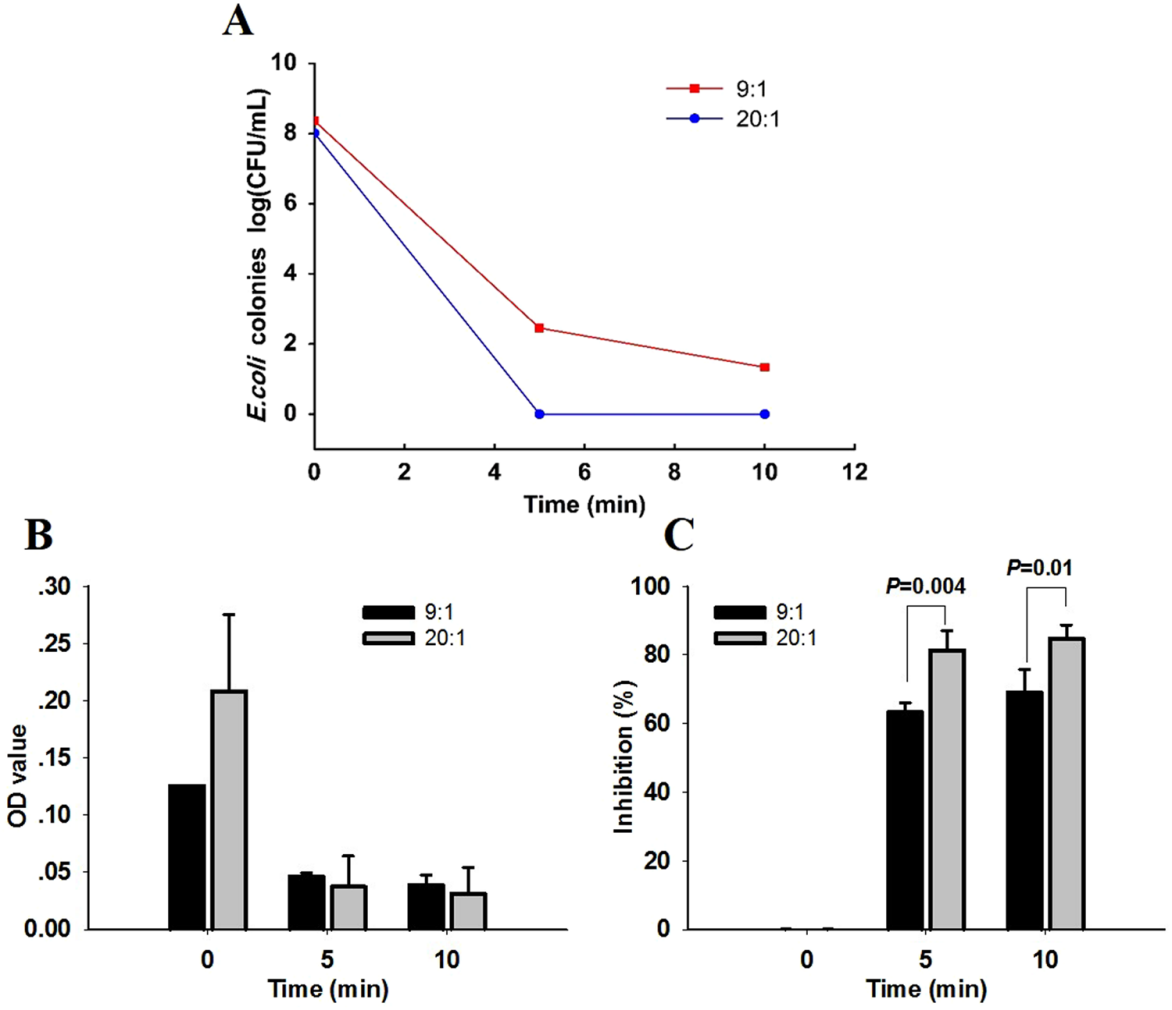
Inhibition of cell proliferation induced by SAEW was determined by colony count and MTT assays. (**A**) Statistical *E. coli* colony data were measured following 9:1 and 20:1 volume ratios under SAEW treatments at 0, 5 and 10 min. (**B**) OD absorbance values and **(C)** cell proliferation were measured following 9:1 and 20:1 volume ratios under treatments at 0, 5 and 10 min (n = 4).

The physiochemical parameters of SAEW were pH 6.40, an ORP of 900 mV, and an ACC of 60 mg/L in all experiments. The SAEW solution reduced the *E. coli* population levels on LB agar medium, which is shown in Fig. 2A. When the volume ratio of SAEW and bacteria solution was 9:1, the colony counts fell from 8.36 ± 0.10 log (CFU/mL) to 2.45 ± 0.07 log (CFU/mL) after being treated by SAEW for 5 minutes, and they fell to 1.33 ± 0.11 log (CFU/mL) after 10 minutes. Moreover, when the volume ratio of SAEW and bacteria solution was 20:1, the *E. coli* was eliminated after only 5 minutes of SAEW treatment. A higher bactericidal effect was observed at the volume ratio of 20:1 than at the 9:1 (*P* < 0.05, n = 3). However, no significant differences were observed between the 5- and 10-minute treatment for the 9:1 and 20:1 volume ratio, respectively (*P* > 0.05).

Colony count was used to estimate the cloning potential of *E.coli* by treating with SAEW. To further elucidate the immediate effect of SAEW on the *E.coli*, MTT assay was used. As shown in Fig. 2B, the OD value was decreased significantly both at the ratio of 9:1 and 20:1. A higher level of *E. coli* inhibition was also observed with the volume ratio of 20:1 at both 5 (*P* = 0.004) and 10 minutes (*P* = 0.01, Fig. 2C). The results of plate dilution colony counting and MTT colorimetric method showed that with an increased volume ratio, the inhibitory effect on *E. coli* was more effective.

### The change of the proportion of living *E. coli* and dead *E. coli* induced by SAEW was detected

**Figure 3:**
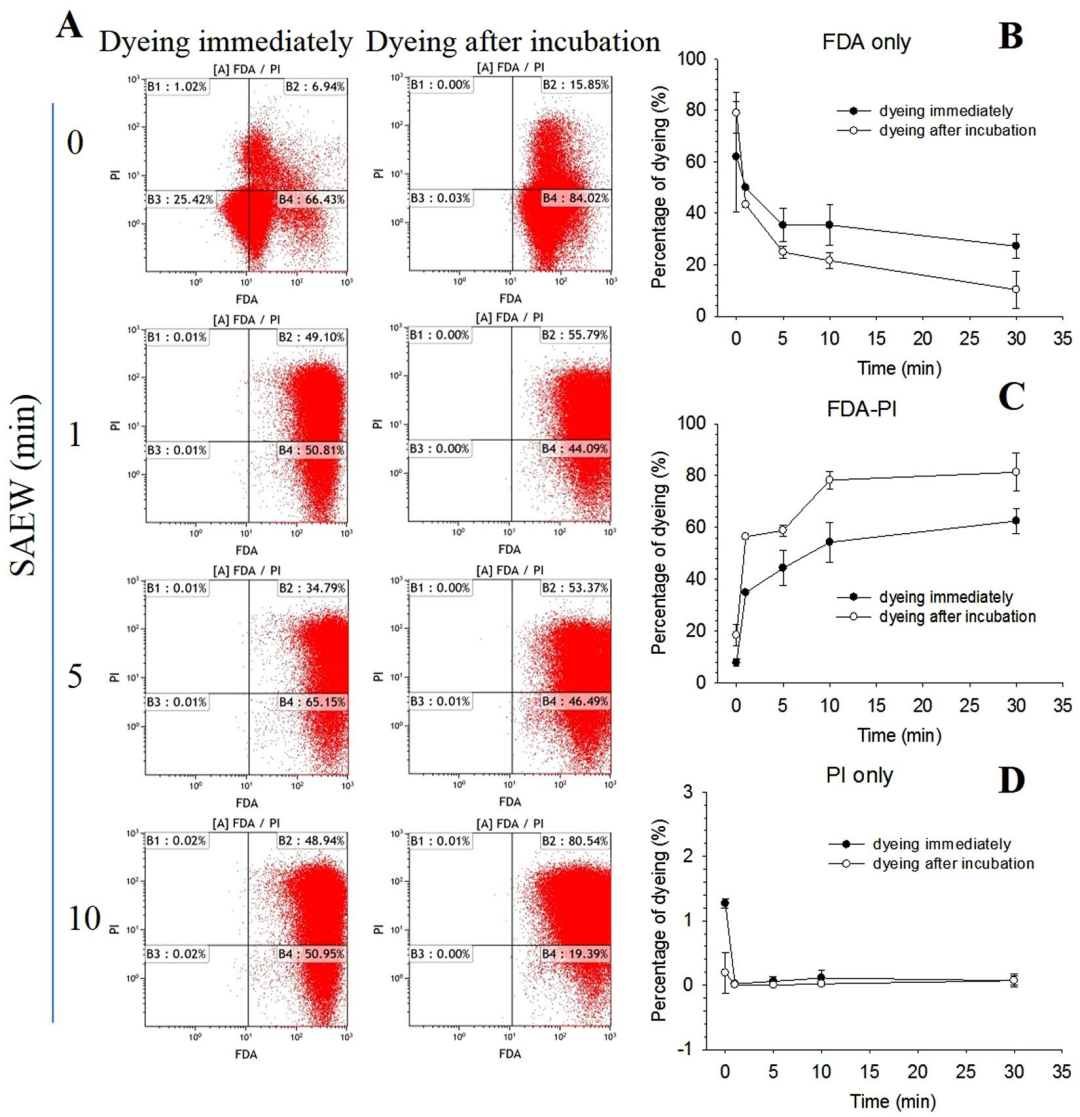
The live-dead proportion of *E. coli* following SAEW treatment was estimated with a FDA-PI fluorescence assay. (**A**) The representative data were measured by flow cytometry under treatment of SAEW with the volume ratio of 20:1 at 0, 1, 5 and 10 min. Cell numbers dyeing with **(B**) FDA (+), (**C**) FDA-PI(+)and (**D**) PI(+) following the volume ratio of 20:1 within 0, 1, 5 and 10 min.

FDA-PI double dye was used to demonstrate the influence process of SAEW on *E. coli* survival state. During the observation duration, the number of cells dyeing immediately with FDA(+), PI(−)decreased gradually (Fig. 3A and B), and dyeing immediately with FDA(+), PI(+) increased gradually by treating ﻿with﻿ SAEW (Fig. 3A and C). These phenomena showed more obviously by dyeing after incubation. Very few cells were stained with FDA(−), PI(+) (Fig. 3A and D) and FDA(−), PI(−).

### SAEW induced cell morphology and permeability changes in the *E. coli*

**Figure 4:**
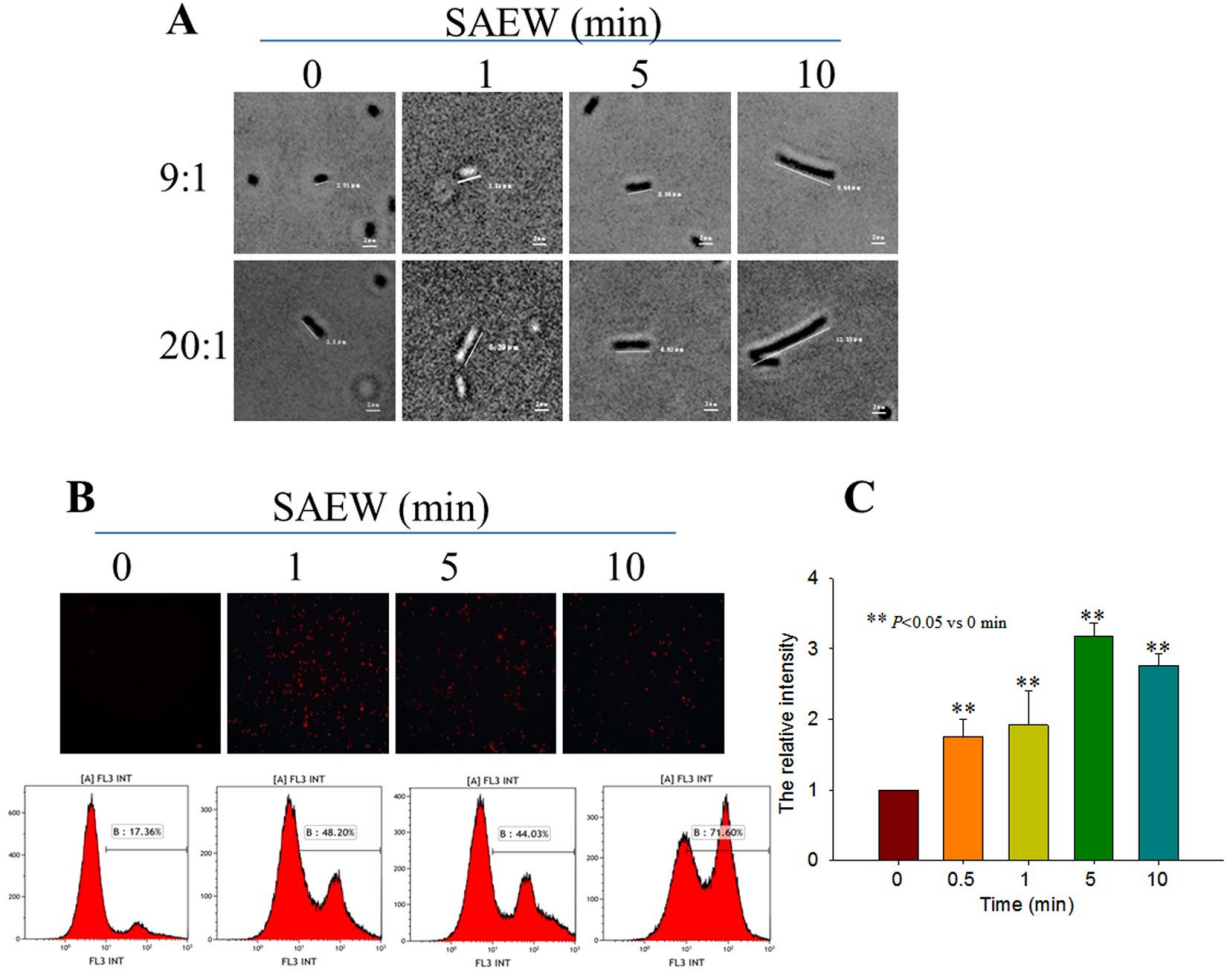
SAEW induced *E. coli* morphology and permeability changes. (**A**) The representative cell morphology data were observed under a microscope. **(B)** The representative cell permeability data were observed under a fluorescence microscope and by flow cytometry. **(C)** The statistical data of the relative PI fluorescence intensity were measured by flow cytometry (n = 4, **P < 0.01 vs 0 min).

The above data indicated that treating of SAEW with less than 5 min led to the viability increase and cell membrane integrity destroy. Next, cell morphological changes and cell permeability were studied. As shown in Fig. 4A, compared with the control condition, SAEW induced cell swelling and bloating, with lengths increasing at 1, 5 and 10 min. The relative PI average fluorescence intensity via flow cytometry detection is shown in Fig. 4B. The bacterial cell membrane permeability was enhanced, and the cells were stained by PI. The relative PI average fluorescence intensities of the *E. coli* treated with SAEW were significantly higher than those in the control group (n = 3, *P* < 0.05). The relative average fluorescence intensity was strongest in the 5 min SAEW treated samples (Fig. 4C).

### SAEW induced reactive oxygen species production by *E. coli*

**Figure 5:**
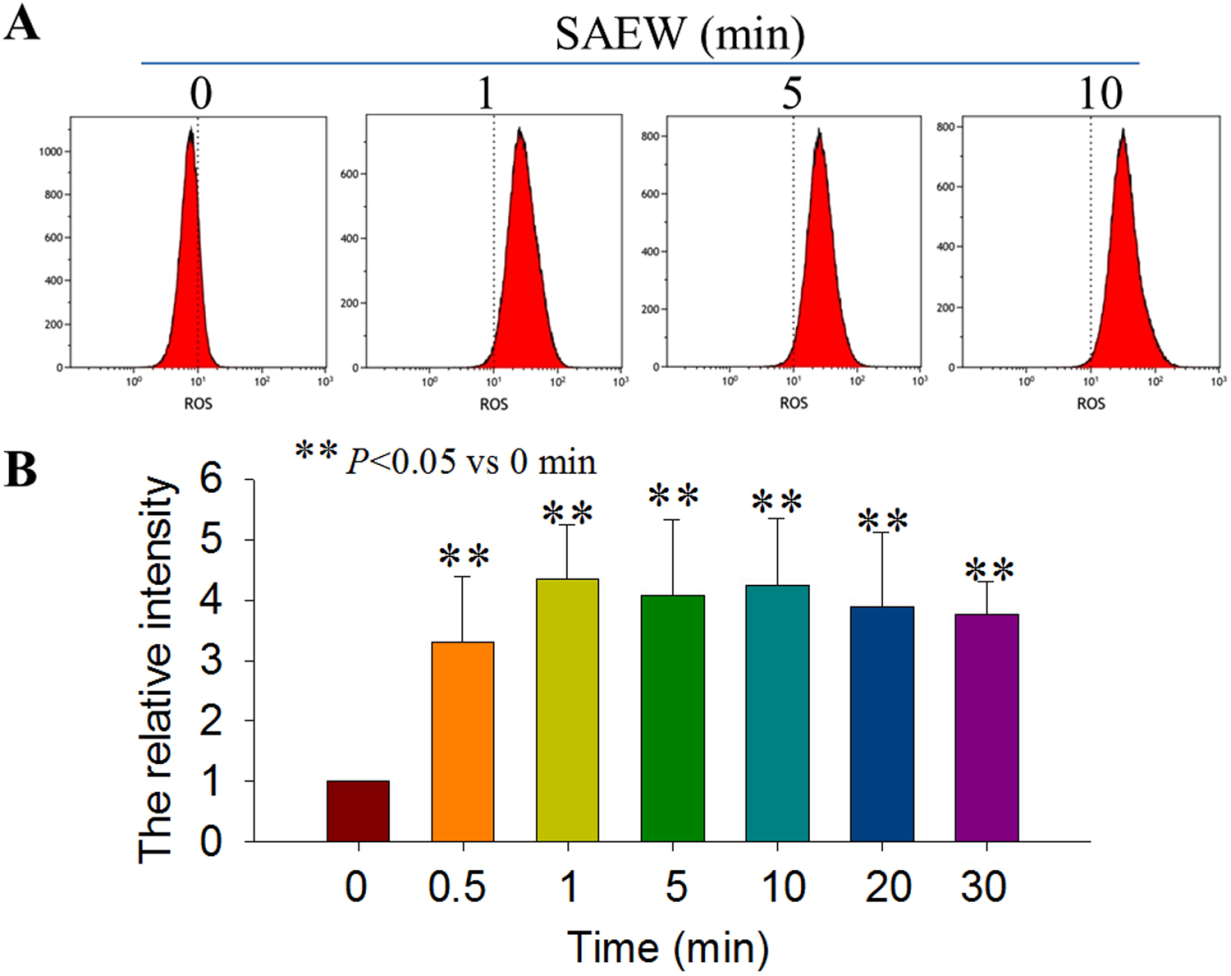
SAEW induced ROS production in *E. coli*, as measured by flow cytometry. (**A**) The representative and (**B**) the statistical DCF fluorescence data were observed following SAEW treatment with the volume ratio of 20:1 (n = 4, **P < 0.01 vs 0 min).

The bacterial DCF fluorescence intensity was proportional to the amount of ROS in the cells^34, 35^. As shown in Fig. 5A, compared with the control conditions, the intracellular DCF fluorescence increased 3–4 fold at 1, 5 and 10 min compared with the 0 min group (n = 3, *P* < 0.05; Fig. 5A and B) following SAEW treatment. These observations indicated that SAEW induced ROS production.

### *E. coli* exhibit characteristic markers of necrosis and apoptosis induced by SAEW

**Figure 6:**
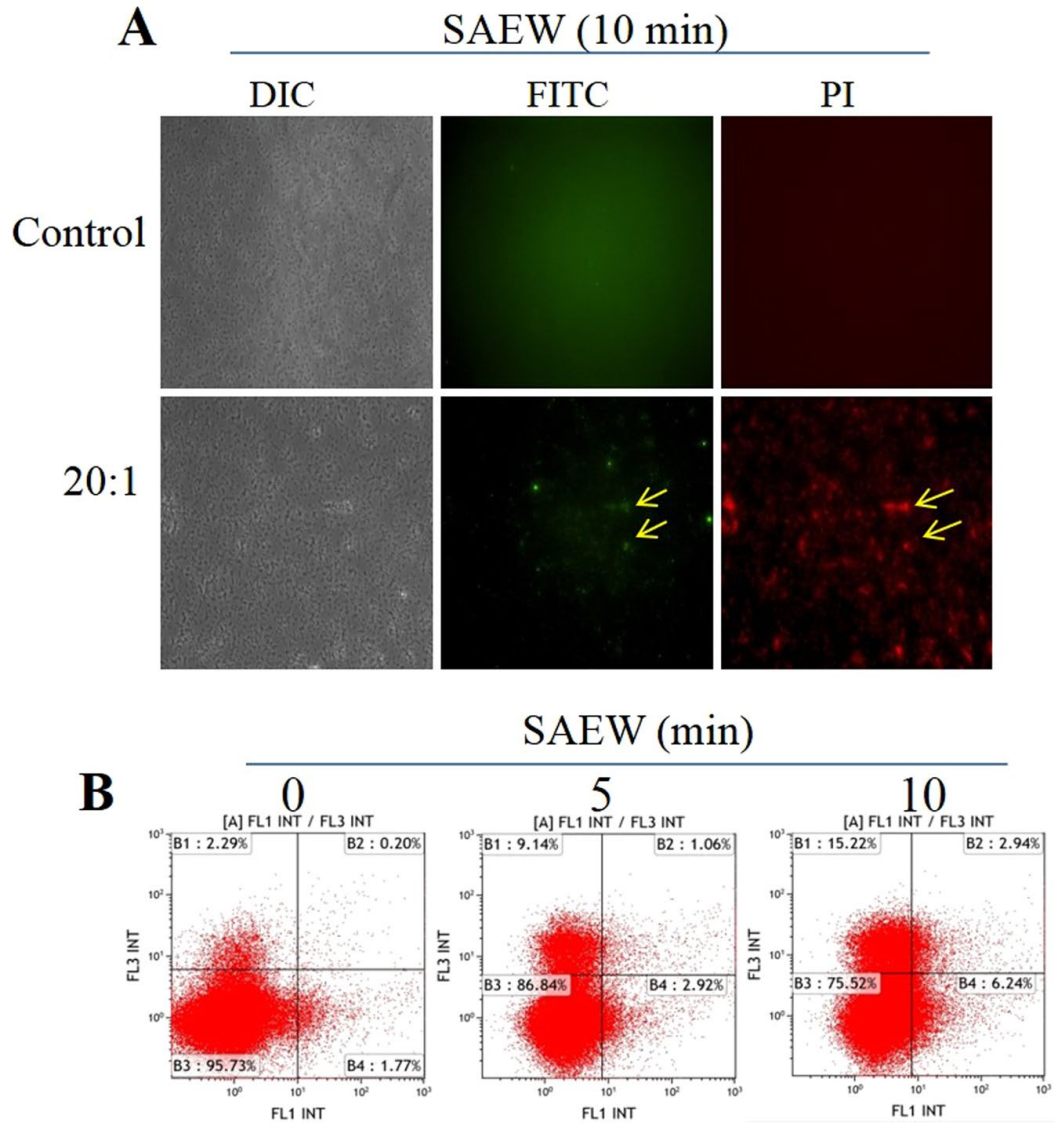
SAEW induced *E. coli* necrosis and apoptosis, as measured with fluorescence microscopy and flow cytometry. (**A**) The representative data of the FITC-PI label were observed with a fluorescence microscope and **(B**) flow cytometry.

Phosphatidylserine (PS) is normally located inside the cell membrane. Externally exposed PS is a typical biochemical marker of apoptosis^36^, which can be observed by Annexin V-FITC/PI apoptosis detection. Dwyer, D. *et al*.^33^ observed this phenomenon when *E. coli* were treated with antibiotics through Annexin V-FITC/PI apoptosis detection. In this study, no obvious fluorescence was observed in control conditions under a fluorescence microscope (As shown in Fig. 6A control). A small proportion of the cells with Annexin V-FITC/PI double dyeing which indicated the apoptosis can be observed (As shown in Fig. 6A yellow arrow). However, most of the cell stained only with PI (red fluorescence) represented that the necrosis or cell damage was the main manifestation induced by SAEW. Figure 6B supplied a little more evidence that SAEW induced apoptosis and necrosis in the bacteria. As shown in Fig. 6B, the apoptosis rate and the necrosis rate increased to around 9% (B2 + B4) and around 15.2% (B2) was induced treatment with SAEW at 10 min, respectively.

## Discussion

In this study, the bactericidal effect of SAEW on *E. coli*, which manifested features of apoptosis and necrosis, was estimated. First, the traditional method of counting colony numbers showed that the bactericidal effect was more obvious at the volume ratio of 20:1 (SAEW to bacteria solution) than at the volume ratio of 9:1. The MTT method is not only a popular colorimetric assay for appraising metabolic activity of mammalian cell but can also be used as a quantitative evaluation of viable organisms^37, 38^. The results also demonstrate highly bactericidal effects of SAEW on *E. coli* within several minutes. It is worth noting that the inhibition of SAEW at the ratio of 20:1 on *E. coli* was incomplete by using MTT assay, while colony formation assay showed a complete elimination. These data manifested that the proliferation potential of a small proportion of *E.coli* by treating with SAEW (20:1) was lost even though they still had mitochondrial succinate dehydrogenase.

Fluorescence-based live-dead assays can be performed with FDA and PI to evaluate viable and dead cells. FDA fluorescence can be converted by esterases that exist in live cells from non-fluorescent FDA. However, PI reaches into the cell and intercalates with the DNA double helix through disordered or dead cell membranes. In this study, the FDA fluorescence decreased and FDA-PI fluorescence increased significantly but completely in the cells that dyed immediately indicated that instantly effect of SAEW may not cause the *E.coli* dead totally, which correlated with the MTT assay. But the potential for growth was stunted with the observation in the cells that dyed after incubation. The results of FDA-PI double dye indicated that the cell esterase activity and cell membrane integrity were damaged by SAEW application^39^.

During the bactericidal process, morphological changes often can be induced by antibiotics or disinfectants^33^. Here, we found that the *E. coli* elongated and were bloated by SAEW. SAEW could change the *E. coli* morphology but still maintain their cell shape, which indicated that the cells may not disrupt or spilt following SAEW treatment. However, the cell shape existence does not imply the cellular function still existence. We found the cell permeability increased, which means the cell membrane integrity was destroyed. In terms of changes of cell membrane permeability, our postulation was verified when the PI fluorescence increased following SAEW treatment. These phenomena can also be observed with different stimuli, such as heat and high pressure sterilization on bacteria with irreversible loss of membrane integrity, as indicated by PI uptake^40, 41^. Wenwei Tang *et al*.^42^ found that electrochemical oxidizing water (EOW) could strengthen membrane permeability, improve the conductivity of suspension and cause leakage of K^+^ and protein out of *Bacillus subtilis* cells. They found that the cell wall and membrane were damaged, which provides a potential explanation for how SAEW may act.

ROS is known to induce apoptotic cell death in various cell types^43^. The bacterial DCF fluorescence intensity was proportional to the amount of ROS in the cells^44^. In this study, the relative ROS contents reached a maximum value around 5 minutes and maintained to 30 minutes. The relative ROS contents in the *E. coli* increased significantly after treatment with SAEW (*P* < 0.05), which produced free radicals, such as superoxide anion (O_2_^**·**−^), hydrogen peroxide (H_2_O_2_), and hydroxyl radical (**·**OH) that could oxidize the lipids, glycolipids, and proteins in the cytomembrane into peroxides^45, 46^. Jeong, J. *et al*.^47^ examined the role of ROS in electrochemical disinfection, and they found that **·**OH was the major lethal species responsible for *E. coli* inactivation in the chloride-free electrochemical disinfection process.

Annexin V-FITC/PI double dye fluorescence is often used to quantify apoptosis^48^. In this study, under a fluorescence microscope, the green fluorescent label by Annexin V-FITC inferred early apoptosis, and the red fluorescent label by PI inferred late apoptosis. The flow cytometry results also demonstrated that SAEW induced apoptotic characteristics in the *E. coli* cells. These results are similar to the conclusions of a study reported by Dwyer, D. *et al*.^33^ SAEW damaged bacteria rather than causing immediate death.

## Conclusion

In a whole, SAEW showed an effective bactericidal effect on the *E. coli* which associated with the physiological and biological changes of apoptosis and necrosis induced by SAEW. Our investigation may further illustrate the disinfection mechanisms and expand the applications of SAEW.

## Materials and Methods

### Preparation and physiochemical property measurements of SAEW

SAEW was produced by electrolysis of 6% HCl solution using a flow-type electrolysis apparatus equipped with an electrolytic cell without a separating membrane between the anode and cathode at a voltage of 220 V (HD-240L, Wangpu Trading Co., Ltd, Shanghai, China). The SAEW was prepared on the day of the experiments and used within 1 h of production. The physicochemical properties of SAEW were determined immediately after generation. The pH and ORP were determined with a multi-functional pH/ORP meter (LE-438, METTLER TOLEDO, Shanghai, China) bearing a pH electrode and an ORP electrode. The available chlorine concentration was evaluated by the standard iodometric titration method^49^.

### Preparation of bacterial suspension

The strain, *Escherichia coli* ATCC25922, was obtained from the China General Microbiological Culture Collection Center (CGMCC). Prior to each experiment, The stock cultures were transferred into 100 mL nutrient broth (Huankai Microbial Sci. & Tech. Co., Ltd., Guangdong, China) and incubated in an incubator shaker (SHA-CA, Honghua Instrument Factory, Jintan, China) for 24 h at 150 r/min, 37 °C. A 5-ml volume of the enriched culture was pooled into sterile centrifuge tube and subsided in a refrigerated centrifuge (5424 R, Eppendorf China Ltd., Shanghai, China) at 5000 rpm, 4 °C for 10 min. The resulting cell pellet was washed twice and resuspended in 5 mL of sterile phosphate buffer solution (PBS) to obtain a final bacterial cell density of approximately 10^9^ CFU/mL which was confirmed by plating 1 mL portions of appropriately diluted *E.coli* suspension on plate count agar plates and then incubated at 37 °C for 24 h.

### Effect of SAEW on microbiota inactivation

The SAEW (pH: 6.40, ORP: 910 mV, ACC: 60 mg/L) used in this study was generated by electrolysis. In SAEW treatment experiments, SAEW and *E. coli* cell suspensions that were prepared as previously described were mixed at ratios of 9:1 and 20:1 for 5 and 10 min. At the end of each treatment time, 1 ml of each sample was transferred to a sterile tube containing 9 ml neutralizing buffer solution (0.5% Na_2_S_2_O_3_ containing 0.03 M phosphate buffer solution) to halt disinfection by SAEW. After 5 min of neutralization, treated and control (untreated) samples were then serially diluted in sterile PBS. Subsequently, viable colonies were enumerated by the plate dilution colony counting and MTT colorimetric methods (Sigma, America).

### MTT (3-[4,5-dimethylthiazol-2-yl]-2,5-diphenyltetrazolium bromide) colorimetric method

MTT that is water soluble, is converted to insoluble formazan by cleavage of the tetrazolium ring by succinate dehydrogenase in the presence of live cells^50^. Since formazan is purple, the change in colour of the solution provides an indication of cellular activity. MTT allows for cellular activity to be quantified by measuring the absorbance of the solution at certain wavelengths^33, 37, 38^. After treatment with SAEW (and PBS as a blank control), samples were centrifuged at 5000 rpm for 5 minutes in a refrigerated centrifuge (5424 R, Eppendorf China Ltd., Shanghai, China). The supernatant was removed and then re-suspended in 1 mL of sterile broth. Bacteria liquid was added into 24-well culture plates (100 μL of each sample per well). Each group was established in three holes. Ten microliters of MTT was added to each well, which was then incubated at 37 °C for 4 h without light. Then, 100 μL of dimethyl sulfoxide (DMSO) was added to each well, and the bacterial liquid was vibrated in an incubator shaker for 10 minutes until the crystalline DMSO dissolved. Afterwards, the absorbance of each sample was measured at an OD of 570 nm with an ELISA instrument. Inhibition rate of *E.coli* proliferation was calculated by OD values.

### Determination of *E. coli* morphologic change

Two hundred microlitres of bacterium suspension was drizzled in the polylysine coated slides in 6-well plates. Then, the suspension was submerged in 2 mL of sterile broth at 37 °C for 3 h to make it stick on the glass for easy observation. The excess broth was then removed, and 4 mL of SAEW (PBS as a blank control) was added to the bacteria for the pre-set times. At each timepoint, approximately 10^6^ cells were collected. The cells were washed once and observed under a fluorescence microscope (ECLIPSE Ti-s, Nikon, Japan).

### Determination of *E. coli* reactive oxygen species content

Reactive Oxygen Detection Kits (Blue skies biotechnology, China) were used for detection of *E.coli* ROS levels by fluorescence probe (DCFH-DA, (2,7-Dichlorodi-hydrofluorescein diacetate)). The fluorescence intensity was proportional to the levels of ROS. After treatment with SAEW (PBS as blank control) for pre-set times, the samples were centrifuged at 5000 rpm for 5 minutes in a refrigerated centrifuge. They were then washed once and resuspended in 200 μL of DCFH-DA working solution and incubated at 37 °C in the dark for 20 minutes. To detect the ROS contents by BD flow cytometry (GALLIOS, BECKMAN COULTER, USA), approximately 10^6^ cells were collected for each sample.

### Fluorescein diacetate (FDA)-propidium iodide (PI) double dye analysis to determine the influence of SAEW on cell activity

The influence of SAEW on cell activity was detected by FDA (West Asia reagent, China) and PI (Blue skies biotechnology, China). FDA is a non-polar, hydrophobic, non-fluorescent esterified compound, which readily permeates the cell membrane and is hydrolyzed by non-specific esterases producing a fluorescein. FDA was used to indicate the presence of active esterase. Meanwhile, PI was utilized to assess cell membrane integrity. Cells with an intact cell membrane and inactive esterase cannot be stained with FDA or PI. After treatment with SAEW (PBS as a blank control) for pre-set times, samples were centrifuged at 5000 rpm for 5 minutes in a refrigerated centrifuge and washed once. Then, the samples were divided into two groups: those that were dyed immediately and those that were dyed after a 4-h incubation. In the immediately dyed group, cells were resuspended in one hundred microlitres of binding buffer. Ten microlitres of FDA working solution and 2.5 μL of PI were added to each sample, which were then incubated in the dark for 10 minutes. In the group that was dyed after the 4-h incubation, cells were resuspended in 1 mL of sterile broth and incubated for 4 h. Then, the samples were centrifuged at 5000 rpm for 5 minutes in a refrigerated centrifuge and washed once before the staining step. Approximately 10^6^ cells were collected for each sample. The treated cells were detected with a BD flow cytometer.

### PI single-staining to analyse the influence of SAEW on membrane permeability

Two kinds of detection methods to determine PI single-staining were utilized: fluorescence microscope observation and flow cytometry.

#### Fluorescence microscope observation

Two hundred microlitres bacterium suspension was drizzled onto polylysine coated slides in 6-well plates. Then, the bacterial suspension was submerged in 2 mL of sterile broth at 37 °C for 3 h so that it would stick on the glass for easy observation. Then, the excess broth was removed, and 4 mL of SAEW (PBS as a blank control) was added to the bacteria for the pre-set times. The cells were washed once, and then 100 μL of PI diluent (100 μL of binding buffer + 2.5 μL of PI) was added to each sample, which were then incubated in the dark for 10 minutes at room temperature. At each timepoint, approximately 10^6^ cells were collected. The cells were washed once and observed under a fluorescence microscope.

#### Flow cytometry

After treatment with SAEW (PBS as a blank control) for the pre-set times, the samples were centrifuged at 5000 rpm for 5 minutes in a refrigerated centrifuge, washed once and resuspended in 100 μL of binding buffer. Then, 2.5 μL of PI was added to each sample, which were then incubated in the dark for 10 minutes. Approximately 10^6^ cells were collected for each sample. The treated cells were detected with a BD flow cytometer.

### Annexin V-FITC/PI apoptosis detection

Apoptosis was detected with an Annexin V-FITC/PI apoptosis kit (Blue skies biotechnology, China). Annexin V is a Ca^2+^-dependent phospholipid-binding protein with high affinity for phosphatidylserine (PS). PS externalization is a relatively early event in apoptosis and occurs before plasma membrane integrity. Therefore Annexin V labeled with fluorescein isothiocyanate (FITC) can be used as a sensitive probe for PS exposure upon the cell membrane. In late apoptotic cells and dead cells, PI is able to pass through the cell membrane to make the cell stained. Thus cells can be distinguished at different stages of apoptosis through Annexin V-FITC/PI apoptosis kits. Furthermore, two kinds of detection methods were utilized to determine apoptosis: fluorescence microscope observations and flow cytometry.

The method to affix the bacteria to slides and treat with SAEW was in accordance with the operating procedure of the *E. coli* morphologic change determinations as mentioned above. The cells were washed once, and then 100 μL of Annexin V-FITC diluent (100 μL of binding buffer + 5 μL of Annexin V-FITC) was added to each sample, which was then incubated in the dark for 20 minutes at room temperature. The cells were washed once followed by the addition of 100 μL of PI diluent (100 mL of binding buffer + 2.5 μL of PI). At each timepoint, approximately 10^6^ cells were collected. The cells were washed again and then observed under a fluorescence microscope.

Flow cytometry: After treatment with SAEW (PBS as a blank control) for pre-set times, the samples were centrifuged at 5000 rpm for 5 minutes in a refrigerated centrifuge, washed once and resuspended in 100 μL of binding buffer. Ten microlitres of Annexin V-FITC was added to each sample. Then, 2.5 μL of PI was added to each sample, which was then incubated in the dark for 10 minutes. Approximately 10^6^ cells were collected for each sample. The treated cells were detected with a BD flow cytometer.

### Statistical analysis

For each treatment, data from independent replicate trials were pooled. The flow cytometry data were analysed with CellQuest software. Data integration and drawing processes were conducted with the Origin Version 8 software (OriginLab Corp., USA). One-way analysis of variance (ANOVA) and Tukey’s test for independent replicates were performed using SPSS 19.0 (Statistical Package for the Social Sciences; SPSS, Inc, Chicago, IL) software. Significant differences were defined at P = 0.05. Data were expressed as means ± standard errors.
